# The total stress approach to martensitic transformations in Ti–Nb-based alloys

**DOI:** 10.1038/s43246-025-00986-x

**Published:** 2025-11-17

**Authors:** Nicole L. Church, Christian E. P. Talbot, Simon M. Fairclough, Nicholas G. Jones

**Affiliations:** Department of Materials Science & Metallurgy, University of Cambridge, Cambridge, UK

**Keywords:** Metals and alloys, Aerospace engineering, Mechanical properties

## Abstract

Metastable β Ti alloys have potential for vibration damping and actuation applications within the aerospace industry due to thermal and mechanical hysteresis. However, variations in transformation parameters, which are also seen to change following thermal or mechanical cycling, significantly limit industrial acceptance. There is a widespread belief that these variations are a consequence of ⍵ phase formation. However, here we provide evidence to show that this is not necessarily the case. Instead, we show how residual stresses and defect structures are crucial to the transformation of these alloys and present an understanding of the mechanism that governs their behaviour. Importantly, we highlight the consequences for the design of new transforming alloys and component geometries, and how current design theories may need to be employed in conjunction with other methods to effectively prevent longer-term changes in behaviour. To this end, we demonstrate how functional properties could be periodically recovered by introducing short intercycle heat treatments and suggest possible next steps for advancing our understanding of these materials.

## Abstract (web summary)

Beta-phase titanium alloys are attractive for vibration damping and actuation, and their phase transformations are believed to be controlled by the omega-phase. Here, residual stresses and defects are found to be important for controlling phase transformations, suggesting that the omega-phase might be less important than commonly thought.

## Introduction

Titanium and its alloys are commercially important to the aerospace sector owing to their high specific strength^1^, good oxidation resistance at low temperatures^2,3^, and ease of fabrication^2^. They currently find application in landing gear assemblies, parts of an aircraft fuselage, and in the fan and low temperature compressor blades of gas-turbine engines^4^. More recently, Ti alloys have also been considered to address other issues within the industry, such as the presence of mechanical vibrations. These vibrations are difficult to predict and control, as they arise from a variety of sources over a wide range of frequencies. Near resonance of the system, large amplification can occur, exacerbating structural fatigue damage^5^. As such, there is a need for vibration-damping systems to mitigate these issues.

Metastable β Ti alloys that can undergo a reversible transformation between the bcc β phase and an orthorhombic α″ martensite, display thermal and mechanical hysteresis. Consequently, this transformation could potentially be used as a mechanism to dissipate the energy of vibrations, or to provide mechanical actuation in aerospace components^6^. This solution is attractive as it could be achieved without complex moving parts, which offers a significant reduction in complexity and weight compared to alternative options^7,8^. These alloys also possess several benefits over ordered transforming systems such as NiTi, as they are typically more processable^9,10^ and more amenable to tuning the transformation behaviour by altering composition^11–14^. However, the consistency and stability of the transformation behaviour in β Ti systems are currently a major barrier to their widespread commercial application.

**Fig. 1:**
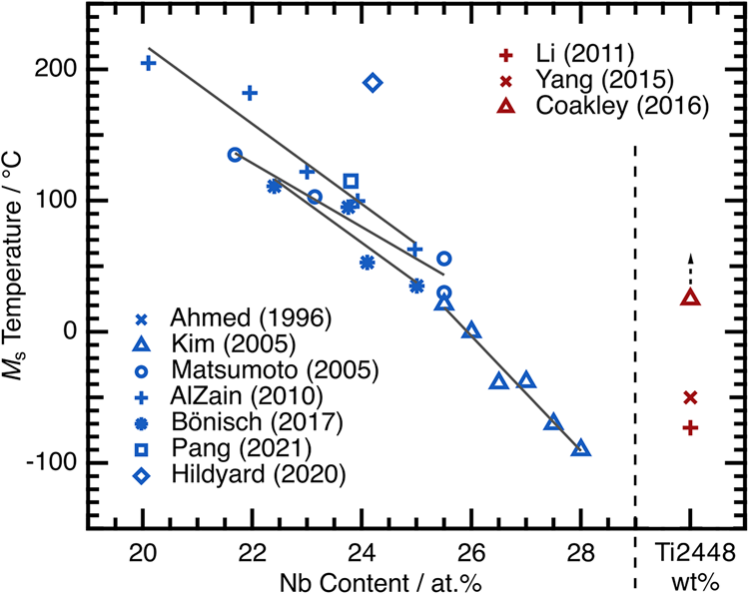
Variation in *M*_s_ temperature as a function of Nb content for a range of Ti–Nb binary alloys. Literature data for a range of Ti–Nb binary alloys. Also shown is the range of reported *M*_s_ temperatures for the commercial Ti–Nb-based alloy, Ti2448. The solid grey lines shown indicate trends from within a self-consistent study. Data from refs. ^16,18–26^.

The thermal transformation is typically characterised by a martensite start temperature, *M*_s_. On cooling below this temperature, the β phase shears to the α″, with this transformation completing at the martensite finish temperature, *M*_f_. On heating, the reverse transformation from α″ to β takes place, starting at *A*_s_ and completing at *A*_f_. In NiTi, the transformation (*M*_s_ to *M*_f_ or *A*_s_ to *A*_f_) usually occurs over a narrow temperature range of ~10–20 °C^11,15^, however, in β Ti alloys, this range can be much larger, although the reasons for this are unclear^16–18^. Furthermore, β Ti alloys can exhibit large variability in the measured value of *M*_s_ even for alloys of nominally identical composition. For example, in the Ti–Nb binary system, *M*_s_ can vary by over 100 °C between studies on the same alloy. When considering studies that do not measure a transformation at all on cooling, this variability exceeds 400 °C. Similar variability can also be observed in the commercial alloy Ti–24Nb–4Zr–8Sn wt% (Ti2448). These inconsistencies are shown graphically as a function of Nb content in Fig. 1, and include comparable data for Ti2448^16,18–26^.

When considering the literature data in more detail, two important observations can be made. First, a general trend of increasing Nb content decreases the *M*_s_ value, which is expected as Nb is a β-phase stabiliser. Second, considering individual studies on a range of binary alloys, the trends observed are approximately linear. The scatter in the data and the variability of *M*_s_ values only arise when comparing the results of different studies, particularly when these linear trends are extended to different Nb concentrations. This cannot be accounted for by variations in the actual composition from the nominal value, as this would be expected to introduce scatter within and between studies. Nor can these discrepancies always be accounted for by variations in the constituent phases present in the microstructure. Instead, this is the first suggestion that variability in the initial condition of the material might be important.

**Fig. 2:**
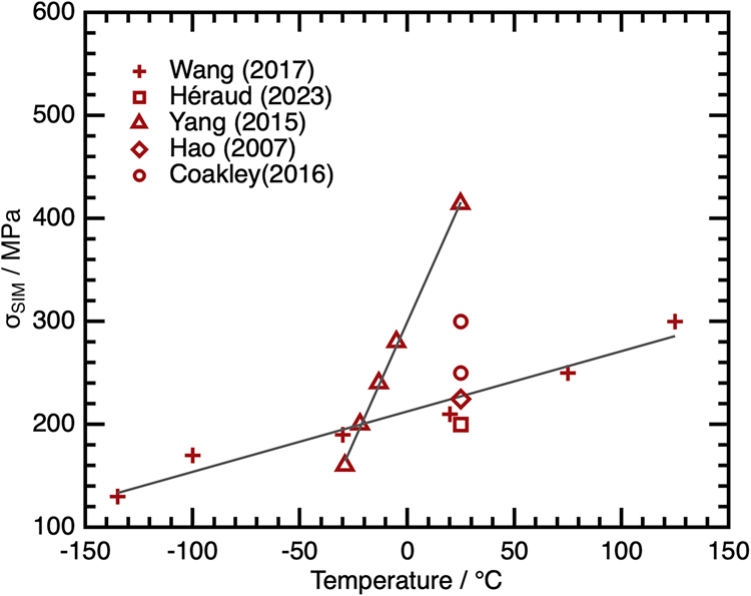
Variation in σ_SIM_ as a function of test temperature for commercial Ti–Nb-based alloy, Ti2448. Literature data for the transformation stress of Ti2448, obtained at different test temperatures. The solid grey lines shown indicate trends from within a self-consistent study. Data from refs. ^20,27–30^.

Similar scatter in transformation-related parameters is evident within the literature when considering the mechanical transformation between β and α″^20,27–30^. This can typically be characterised by a critical stress, σ_SIM,_ which must be exceeded to shear the β lattice. σ_SIM_ is expected to vary with temperature according to the Clausius–Clapeyron relationship, decreasing linearly when tested at lower temperatures^31^. However, when considering literature data on Ti2448, discrepancies in σ_SIM_ of over 200 MPa can be observed even at a consistent test temperature. These data can be visualised in Fig. 2. Again, when considering individual studies, the trends observed do appear to be linear and obey the Clausius–Clapeyron relationship. However, between studies, the gradients of those trends vary dramatically. This would not be expected from conventional theory^32^, as for a given alloy composition, the volume change and entropy change on transformation are not expected to vary between studies. Therefore, the different gradients observed are non-physical without other factors influencing the transformation. This is the second indication that there must be something fundamentally different in the condition of the alloys used between studies.

Measurement of *M*_s_ and σ_SIM_ involves thermally or mechanically cycling the alloy, respectively. However, in both cases, it has been reported that the key transformation parameters change between the first and second cycle^24,28,33^. These observations reaffirm the position that variability in composition alone cannot be responsible for the discrepancies. Importantly, the use of transforming alloys in the aerospace sector has been critically limited by the change in both thermal and mechanical behaviour on cycling and an inadequate understanding of the underlying mechanics.

The evolution of the ⍵ phase is routinely considered responsible for the variation in transformation behaviour between samples of different microstructural conditions and thermal histories. Indeed, many studies have shown that ⍵ it can have a direct influence on the martensitic transformation, and therefore can account for some of the variability within the literature^34,35^. However, some alloys, including Ti2448, are thought to be more resistant to ⍵ formation due to the presence of a high volume fraction of Sn^29,36^. As such, our work to date has challenged the role ⍵ it has in governing the behaviour of Ti–Nb alloys, and instead proposes that there must be an additional mechanism involved^37,38^.

Recently, we have shown through a combination of in situ and ex situ experiments that the macroscopic applied load may not be the sole driving force for a stress-induced transformation^37,39,40^. Here, we use new data to bring different aspects of this theory together for the first time, highlighting the applicability of this understanding in the wider context of transforming systems. Critically, we provide definitive evidence to show that ⍵ it cannot be responsible for the wide variability in transformation behaviour in this alloy.

## Results

### The influence of thermal processing on the microstructure and *M*_s_ temperature

**Fig. 3:**
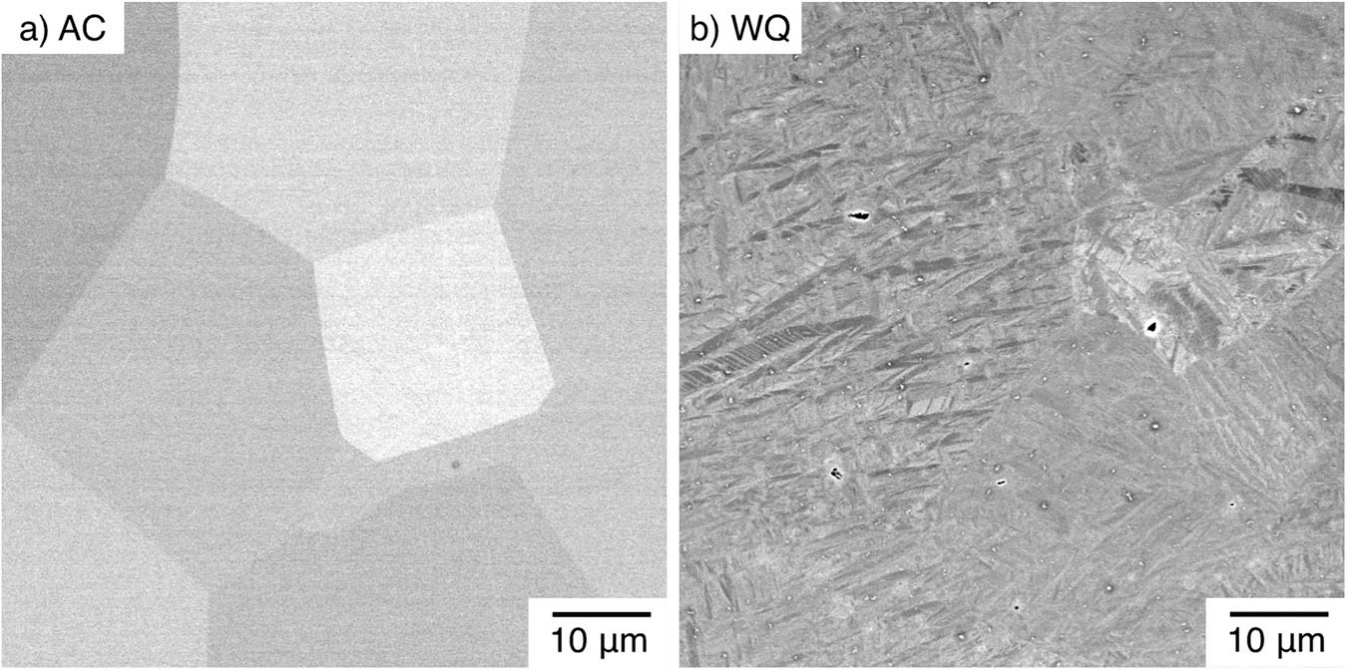
BSE micrographs showing the constituent phases present in each sample following a heat treatment of 900 °C and then cooled at different rates. Showing **a** the AC condition and **b** the WQ condition.

Samples were subjected to identical heat treatments at 900°C and cooled at two different rates. One was air cooled (AC), and the second was water quenched (WQ). Micrographs were obtained for the two microstructural conditions, with these data shown in Fig. 3. In the AC condition, recrystallised grains of the β phase could be observed, with no evidence of any other phase. In the WQ condition, recrystallised β grains were present of similar size and morphology to the AC condition. However, within these β grains, high aspect ratio acicular features could also be observed, consistent with the presence of α″ martensite.

**Fig. 4:**
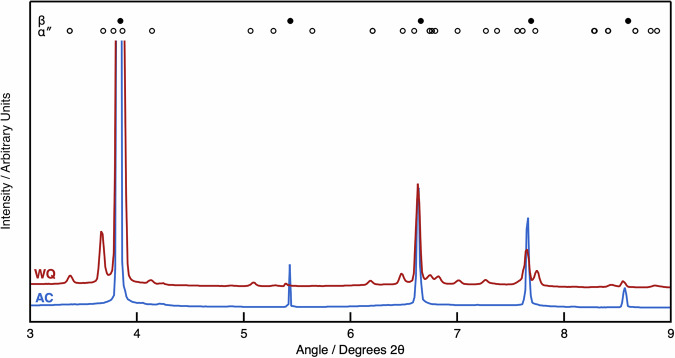
sXRD data highlighting the different phases present in samples of Ti2448 cooled at different rates. Shown are the WQ (red) and AC (blue) conditions.

To confirm the phases observed in the micrographs, synchrotron X-ray diffraction (sXRD) data were acquired in the initial condition, with these data presented in Fig. 4. In the AC condition, strong reflections consistent with the bcc β phase were observed. In the WQ condition, peaks corresponding to the β phase were observed; however, there were additional peaks present, consistent with the α″ martensite. Therefore, the AC condition must have an *M*_s_ temperature below 0 °C, whereas the WQ material must have an *M*_s_ above 0 °C. This suggests that the difference in initial cooling rate was sufficient to change the relative stability of the β and α″ phases.

**Fig. 5:**
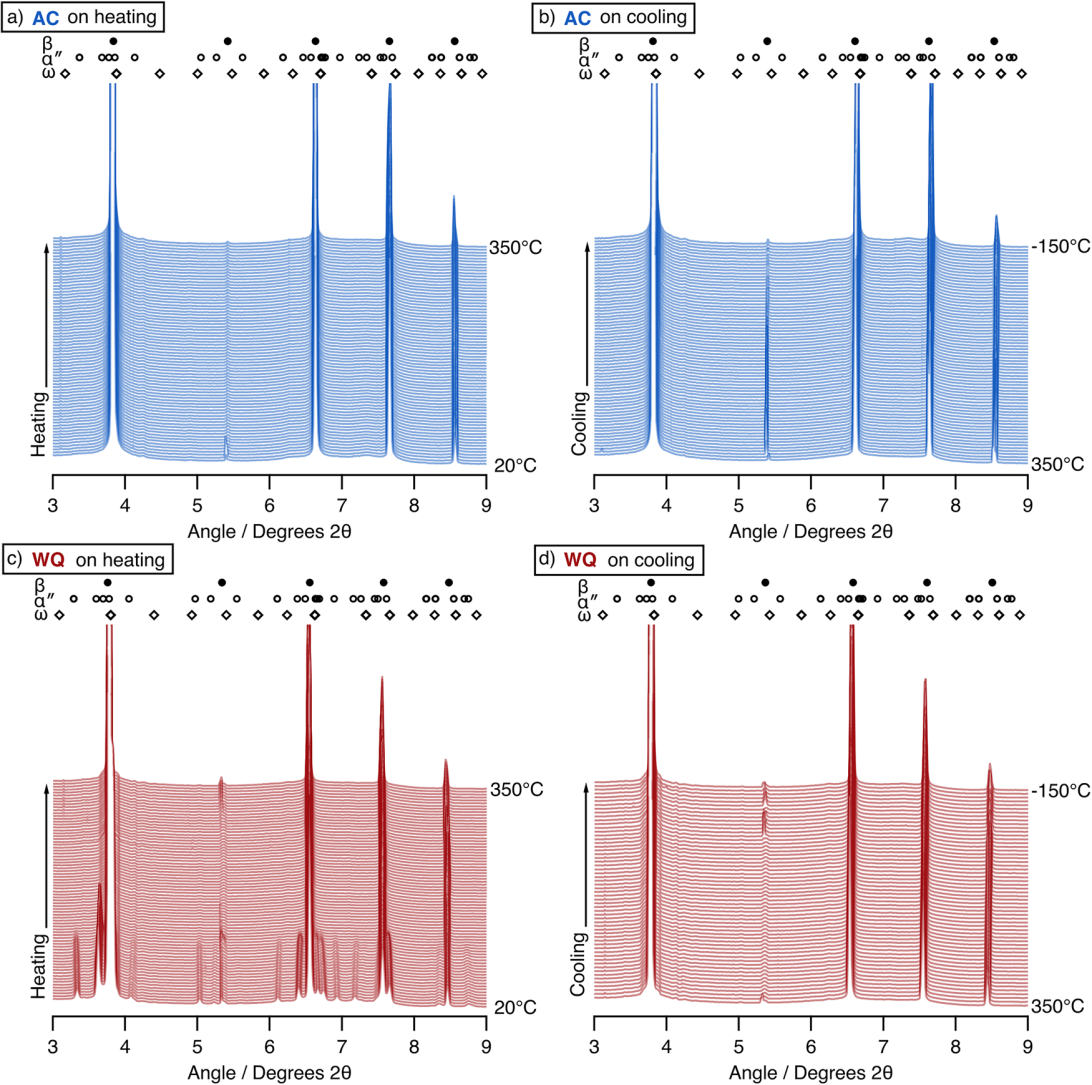
Heating and cooling of Ti2448 in the AC and WQ conditions. The markers highlight the expected locations of β and α″ and ⍵ reflections. **a** Heating the AC condition. **b** Cooling the AC condition following the heating step. No formation of the α″ phase is seen. **c** Heating the WQ condition, showing the reverse transformation from α″ to a single-phase β microstructure. **d** Cooling of the WQ condition following the heating step, showing no formation of α″.

To measure the *M*_s_ temperature of the alloy in the two different conditions, samples in the AC and WQ conditions were first heated to 350 °C in situ, to exceed *A*_f_ and ensure a single-phase β state. The samples were then cooled to −150 °C. The sXRD diffraction patterns on heating and cooling are shown in Fig. 5. On heating the AC condition sample, the β phase peaks shifted slightly to lower 2θ values, consistent with thermal expansion of the lattice. However, there was no evidence of any phase changes in the material. On cooling, the 2θ peak position shifted to slightly higher values as the lattice contracted, with no evidence of any formation of α″. As such, *M*_s_ in this sample condition must be below −150 °C. A small amount of ⍵ phase evolution was observed, with the largest peak intensity and hence highest volume fraction at −150 °C.

In the WQ condition, the initial diffraction patterns contained reflections from both the β and α″ phases. On heating, the reflections from the α″ martensite decreased in intensity, until they were no longer discernible from the baseline at ~135 °C. As such, prior to cooling, the material was in a single-phase β condition, comparable to the AC material. On cooling, the α″ did not reform, even down to −150 °C. This is perhaps a surprising observation and suggests that heating and cooling removed the effect of the initial rapid quench. Formation of the ⍵ phase could again be observed on cooling, with the largest volume fraction inferred to be at −150 °C.

The ⍵ phase exists in two forms, which are crystallographically identical, but can be distinguished based on their composition and method of formation. The athermal ⍵ (⍵_ath_) is compositionally identical to the parent β and forms via a planar collapse of the β structure when cooled from the β phase field. This occurs below the ⍵ start (⍵_s_) temperature, with minimal to no thermal hysteresis observed when the transformation reverses on heating^22^. In contrast, isothermal ⍵ (⍵_iso_) forms as a Ti-rich precipitate at elevated temperatures between ~100 °C and 500 °C^41^ and relies on diffusion. As such, the observation of ⍵ formation on cooling in these materials, with the highest volume fraction observed at the coldest temperature, signifies ⍵_ath_.

**Fig. 6:**
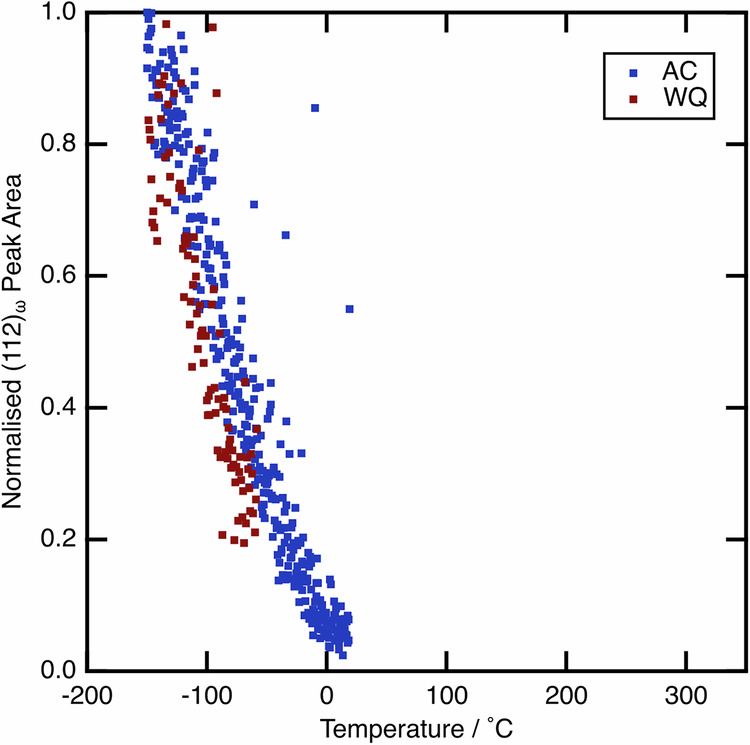
The evolution of the (112)_⍵_ peak area as a function of temperature, when cooling from 350  °C. Areas have been normalised to the value at −150 °C in each case. Both AC and WQ conditions are shown, and are seen to evolve from the same temperature of ~25 °C.

As such, the ⍵_s_ temperatures were measured for both the AC and WQ conditions on cooling from 350 °C to −150 °C, by fitting the peak area of the strongest ⍵ phase reflection and using this as a proxy to volume fraction. These data are given in Fig. 6, where it can be seen that ⍵_s_ is identical for both conditions, at around 25 °C. As such, ⍵_ath_ cannot be responsible for the difference in the observed behaviour. ⍵_iso_ was not observed to form at elevated temperatures in the data presented in Fig. 5, and, given that both samples were subjected to identical thermal profiles, it is also unlikely to be responsible.

### Thermal cycling behaviour—a dynamic microstructure

**Fig. 7:**
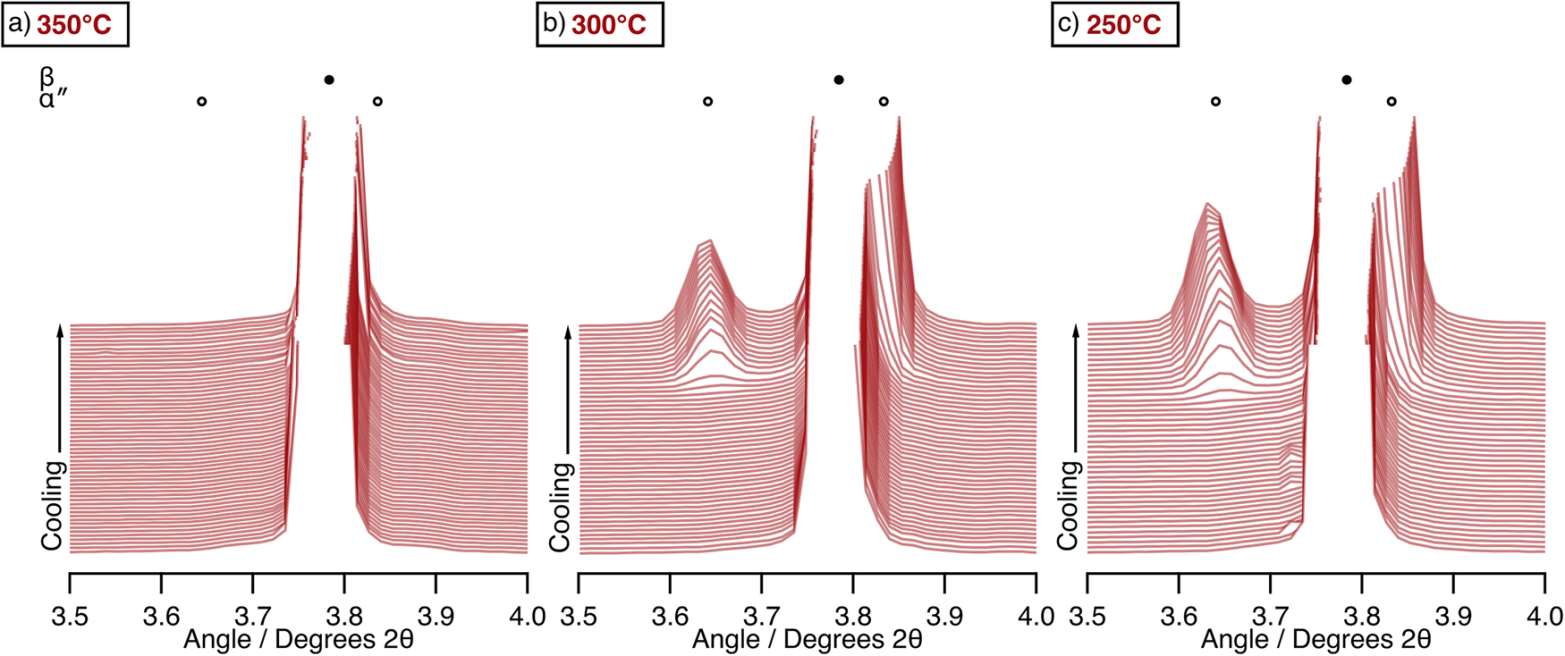
The effect of cooling samples in the WQ condition to −150 °C following heating to different peak temperatures. The data shown is cooling from peak temperatures of **a** 350 °C, **b** 300 °C, and **c** 250 °C. The markers highlight the expected locations of β and α″ reflections. α″ formation is evident only in (**b**, **c**).

Similar heating and cooling cycles were repeated on new WQ samples with varying peak temperatures of 300 °C and 250 °C. The peak evolution on cooling is shown alongside the data when heated to 350°C in Fig. 7. As described above, when cooling from 350 °C, there is no formation of α″ and hence *M*_s_ must be below −150 °C. However, when cooling from 300 °C, an α″ peak did evolve, indicating that *M*_s_ is at a higher temperature. When cooling from 250°C, the α″ peak evolved from a higher temperature still and reached a greater final peak intensity, suggestive of a larger total volume fraction. These data indicate that the peak temperature of the thermal cycle influences the observed transformation behaviour and show that the variations in behaviour are not simply an effect of cooling rate.

**Fig. 8:**
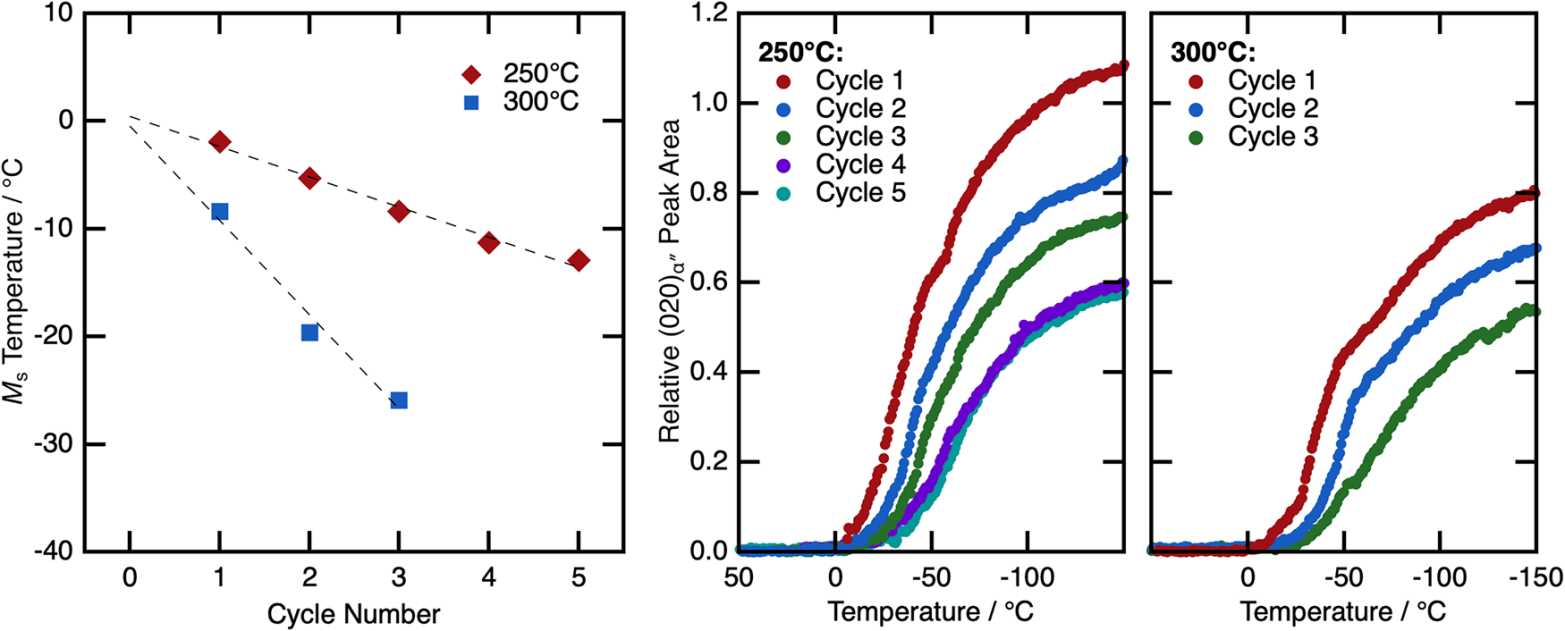
Change in *M*_s_ temperature of the WQ condition as a function of cycle number when heated to both 300 °C and 250 °C. Data shown includes the measured *M*_s_ temperature (left) on cycling between the peak temperature and −150 °C. The peak area as a function of temperature is also shown on cooling for each cycle for peak temperatures of (centre) 250 °C and (right) 300 °C.

The same samples were cycled multiple times between the peak temperature and −150 °C. The area of the (020)_α″_ peak, at ~3.65°2θ, was monitored, and the temperature at which the peak began to be discernible from the baseline was determined to be the *M*_s_ temperature of the material. These data are shown in Fig. 8, and highlight the dynamic nature of the microstructure.

In both cases, *M*_s_ decreased in a linear fashion with each thermal cycle. However, when heated to the higher peak temperature of 300 °C, the rate at which *M*_s_ decreased was greater, which suggests that it is the heating portion of the cycle that is governing the observed behaviour. When considering how the relative volume fraction of the peaks evolves on cooling in the first cycle, it is apparent that the volume fraction of α″ saturates close to −150 °C. This is unexpected, as there is still a significant volume fraction of β remaining in the microstructure at this temperature (Fig. 7) and hence suggests that *M*_f_ is not reached. On cycling, the peak evolution shows a very similar shape. The initial formation of α″ can be seen to be suppressed to lower temperatures with each cycle, however, there is a lower overall volume fraction of martensite, and the same apparent plateau in α″ formation close to −150°C. The decrease in α″ volume fraction highlights that the relative stabilities of the β and α″ phases at any given temperature are being changed. However, the plateau in α″ formation highlights that the transformation to α″ is not merely “shifted” to lower temperatures. Instead, the range of temperatures over which martensite is able to form is being reduced.

### Characterising the mechanical response of samples with different *M*_s_ temperatures

**Fig. 9:**
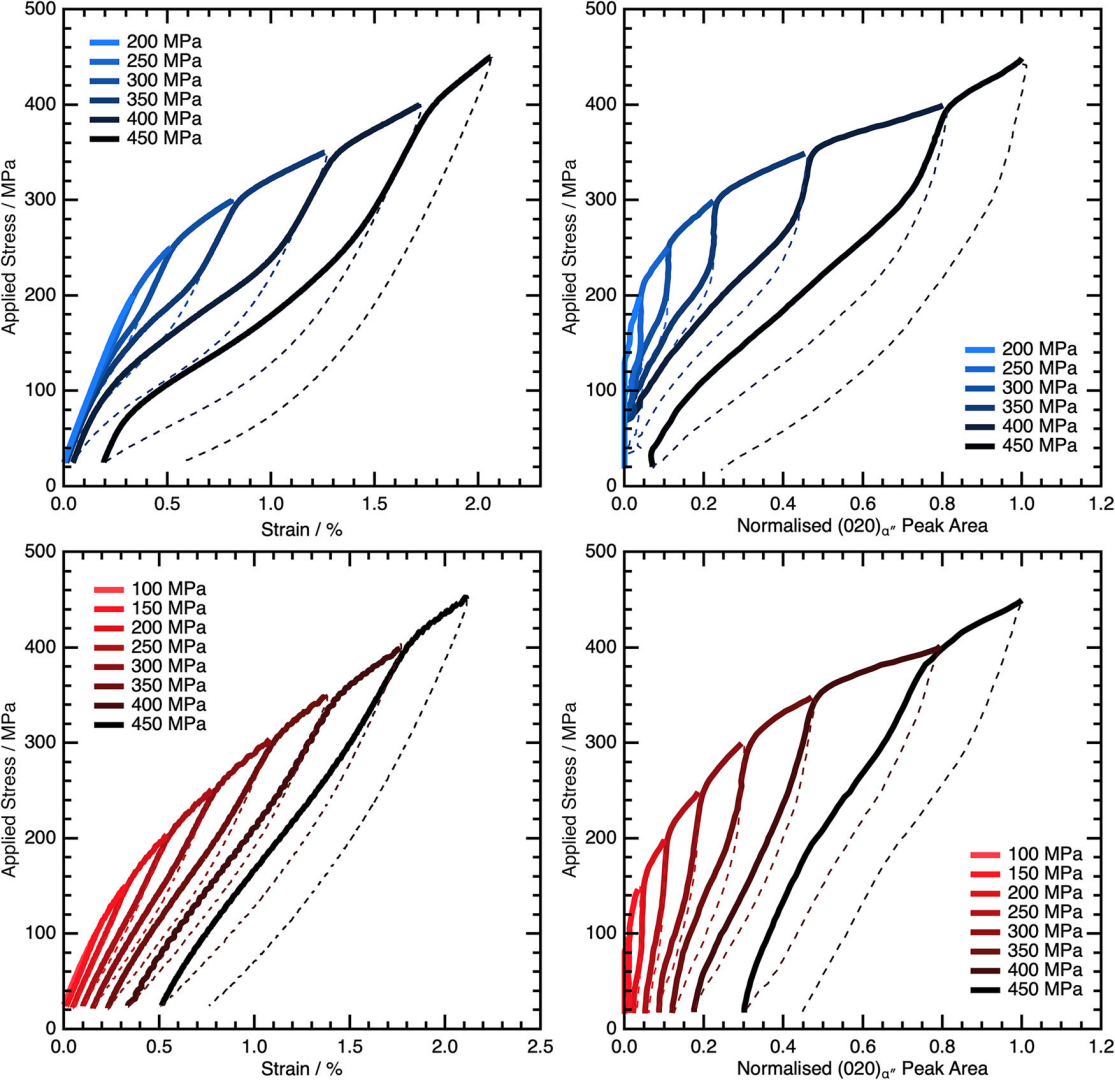
Cyclic loading and unloading to incrementally higher stresses for both the AC and WQ + 300 °C conditions. Both ex situ (left) mechanical data showing measured strain and in situ (right) data showing α″ peak evolution are given on loading and unloading for both the AC (top) and WQ + 300 °C (bottom) conditions. Data for the AC condition were previously published in ref. ^17^.

To assess the effect of the initial condition on the mechanical response of the alloy, incremental stress-strain tests were performed on the AC material, and the WQ material heated to 300 °C and cooled to room temperature, as shown in Fig. 9. This additional heating step was shown in Fig. 8 to depress the *M*_s_ temperature to approximately −10 °C. This ensured the same microstructure was being assessed in each case, but for material conditions with different *M*_s_ values.

In the AC condition material (Fig. 9: blue data, top), the ex situ mechanical response (left) is linear-elastic until approximately 150 MPa, at which point there is a deviation from linear behaviour. This correlates with the onset of the martensitic transformation in the in situ data (right), where it can be seen that there is an increase in the peak area and hence volume fraction of α″ when loading above 150 MPa. On unloading, the transformation is fully reversible and all the α″ transforms back to β, recovering the applied strain. On subsequent cycles, the transformation to α″ begins at a lower stress than in the previous cycle. This has been reported before within the literature and is termed functional fatigue behaviour^28,40,42^. On reaching the same volume fraction of α″ as the previous cycle, the transformation appears to cease, with continued loading resulting only in linear-elastic deformation of the two-phase microstructure. This proceeds until the stress exceeds that of the previous cycle, at which point there is a second decrease in gradient. This is again correlated with an increase in the α″ volume fraction. The transformation remains fully recoverable until a peak stress of 400 MPa is exceeded. On unloading from this stress, there is residual strain in the ex situ data, and a small volume fraction of α″. This change in mechanical response cannot be attributed to the ⍵_iso_ phase, as the temperature remains unchanged during the test.

When considering the WQ + 300 °C material (Fig. 9: red, bottom), the macroscopic tensile response appears to be relatively similar to the AC condition. The sample initially deformed in a linear-elastic fashion up to a stress of ~80 MPa, at which point there was a deviation from linear behaviour. However, this highlights that the WQ + 300 °C material has a lower transformation stress despite being in nominally the same microstructural starting condition. On unloading, there was limited recovery of the α″, with some martensite and residual strain being present even after the first transformation cycle. On loading to higher peak stresses, the transformation stress reduced, consistent with functional fatigue behaviour. As with the AC material, the same two-stage loading behaviour was observed, whereby a sharp increase in the volume fraction of α″ could be seen once the stress from the previous cycle was exceeded.

**Fig. 10:**
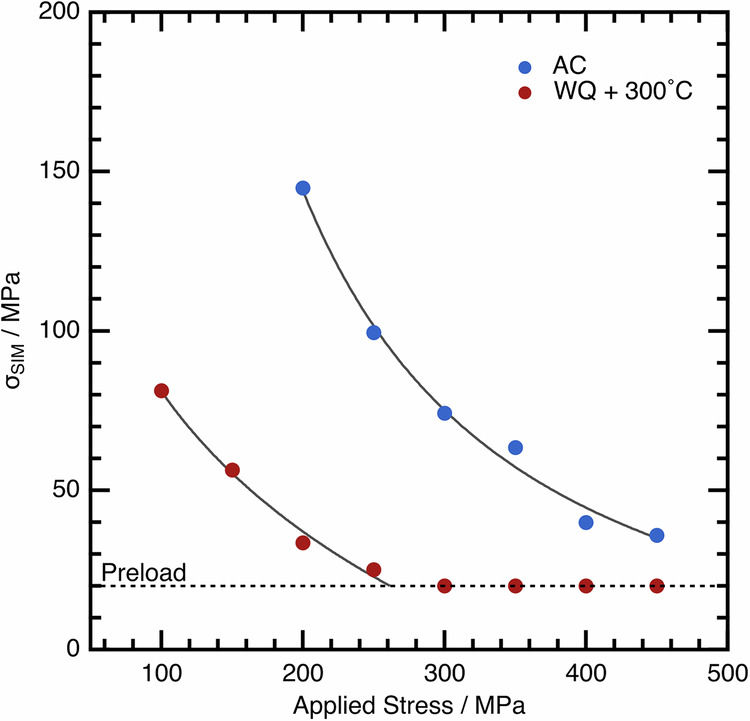
The decrease in σ_SIM_ with each cycle of the incremental stress test. Data are shown for both the AC and WQ + 300°C conditions.

The transformation stress was measured from the in situ data for each cycle, by evaluating the stress at which the α″ peak area first started to increase. This is displayed in Fig. 10, with the provided trend lines used as a guide for the eye. The initial σ_SIM_ differs by ~70 MPa between the two samples, which is in good agreement with other literature observations that show more than twice this difference in σ_SIM_ between WQ, and cold-rolled material in a related alloy^22^.

Both conditions studied here show a decreasing trend in σ_SIM_, which appears to be more gradual for the WQ + 300 °C condition. However, this decrease in σ_SIM_ is limited by the preload on the material. This nonetheless suggests that despite starting with initially very different values of σ_SIM_, they are slowly converging towards similar behaviour.

**Fig. 11:**
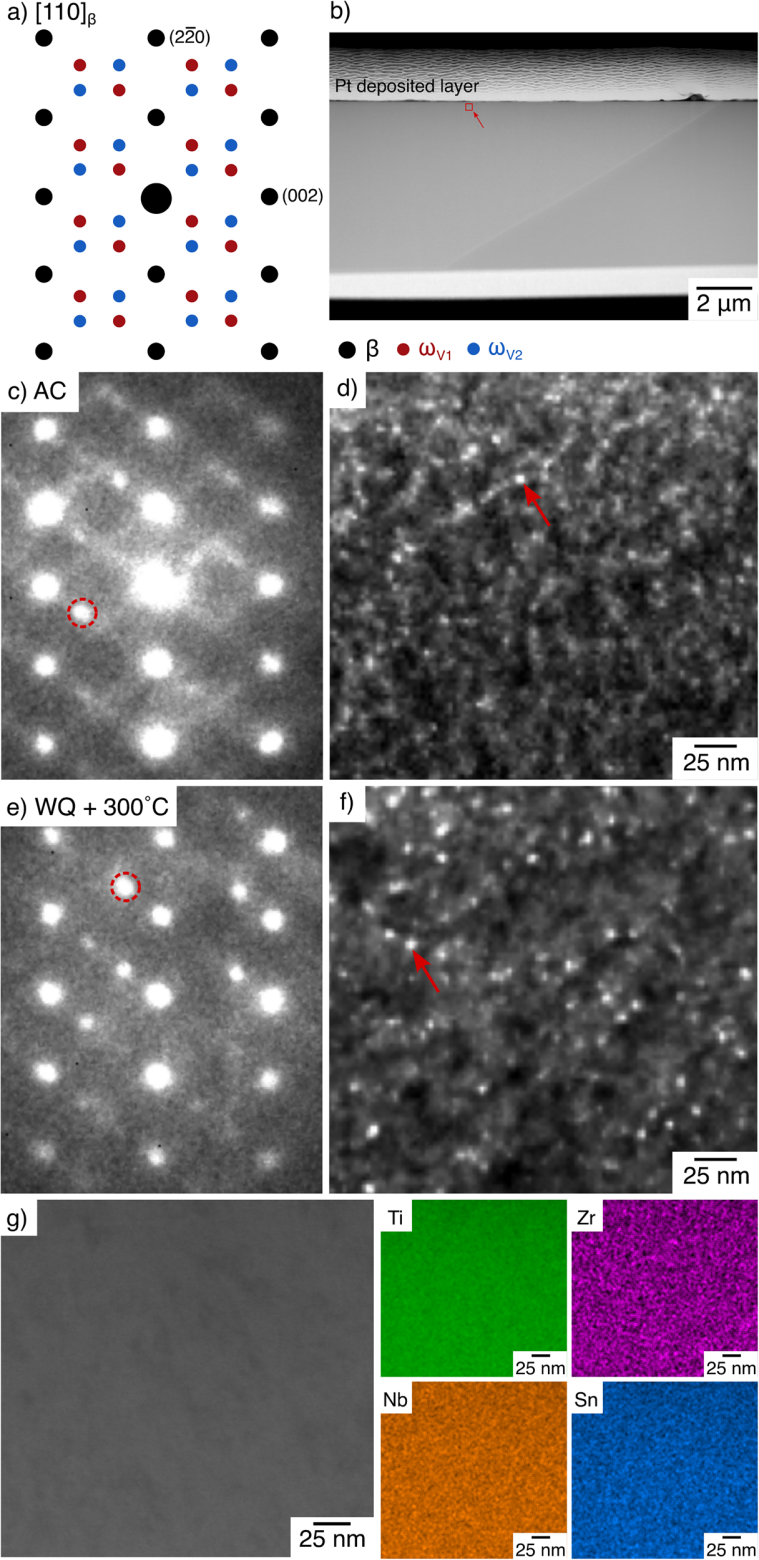
TEM data for the ⍵ phase present in both the AC and WQ + 300 °C conditions. **a** Expected locations of the β and ⍵ phase reflections along [110] of the β phase. **b** Low magnification image of the WQ + 300 °C FIB lamella highlighting the region of interest. **c** A [110] diffraction pattern of the AC condition taken from a region containing ⍵ (region indicated in **d**)). **d** Virtual DF image for the AC condition generated from a virtual aperture around the indicated reflection in **c**). **e**, **f** Comparable data to (**c**, **d**) but for the WQ + 300 °C sample. **g** HAADF STEM and accompanying EDX data of the region shown in **f**), highlighting the lack of compositional segregation due to the ⍵ phase.

Transmission electron microscopy (TEM) focused ion beam (FIB) foils were taken from both the AC and WQ + 300 °C samples, to investigate whether any ⍵_iso_ formation had occurred within these samples. The associated data is shown in Fig. 11.

The lamella for the WQ + 300 °C condition is shown in Fig. 11b, highlighting the area used for subsequent data acquisition. An equivalent area was also considered for the AC condition. Diffraction patterns were obtained at each pixel location by scanning across the same surface. These data are displayed in Fig. 11c, e for both the AC and WQ + 300 °C conditions in a region containing a ⍵ phase precipitate. Reflections consistent with the β phase and an ⍵ phase variant can be seen, confirming a positive identification (c.f. Fig. 11a) of this nano-scale phase. This is in agreement with Fig. 6, which demonstrated ⍵_ath_ formation from just above room temperature in both conditions. The indicated reflections were used to construct virtual dark field (DF) images, shown in Fig. 11d, f. These images suggest a similar size and volume fraction of ⍵ particles within the β matrix. To confirm that the ⍵ was compositionally indistinct from the matrix, a high angle annular dark field (HAADF) image and associated scanning transmission electron microscopy (STEM) energy dispersive x-ray spectroscopy (EDX) data were obtained and are presented for the WQ + 300 °C sample in Fig. 11g. Whilst the small size of the ⍵ phase makes unambiguous identification challenging, no compositional segregation was observed for any element, to or away from the ⍵ phase particles. This suggests that the ⍵ phase present was the ⍵_ath_ form.

**Fig. 12:**
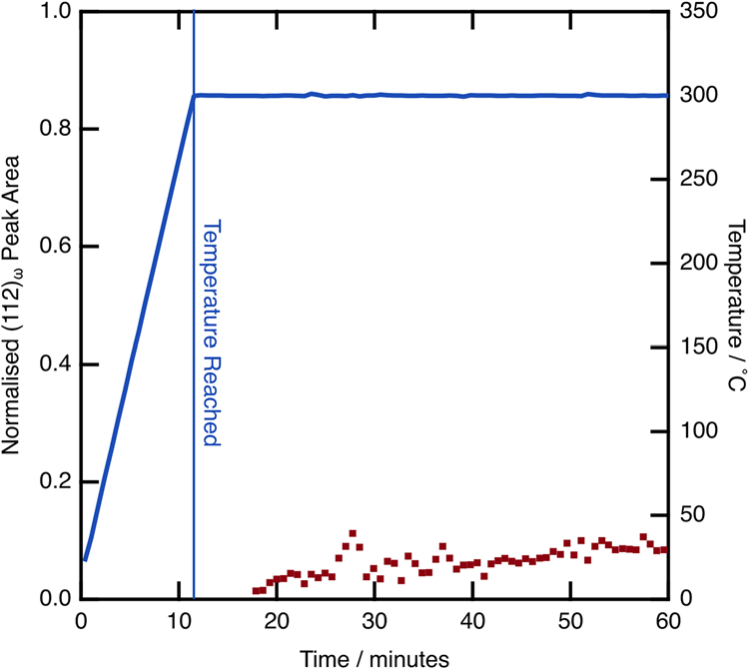
⍵ peak area evolution during the first hour of an isothermal age of a WQ sample at 300 °C. These data include the initial period of heating to the set temperature. The isothermal ⍵ peak evolution is shown, with values normalised to the peak area at the end of the 8 h age. The ⍵ peak is first discernible from the baseline 7 min after the 300 °C set temperature is reached.

To further support these observations, sXRD data were collected during an 8-h isothermal age at 350 °C. This provided information about the susceptibility of this alloy to ⍵_iso_ formation. The peak area of the most intense ⍵ reflection was fit for the diffraction pattern acquired after 8 h, and then this fit was backpropagated to find the onset of precipitation. The first hour of the test is given in Fig. 12, including the heating step to the set temperature.

No ⍵_iso_ formation can be observed on heating, which is consistent with the data presented in Figs. 5 and 11. During the isothermal age, the precipitation ⍵ only started 7 min into the age. As no samples spent this long at elevated temperature (with no dwell period set for any of the heating ramps performed in this work). When considering this observation alongside the results from Figs. 5 and 11, these data suggest that there was not sufficient time for ⍵_iso_ formation.

As such, the combination of Figs. 11 and 12 conclusively shows that ⍵_iso_ cannot be responsible for the discrepancies in behaviour observed between the samples with different thermal histories, nor can it be responsible for the changes in transformation behaviour as a result of thermal cycling, such as in Figs. 5 and 7.

### Restoring functional properties via intercycle heat treatments

**Fig. 13:**
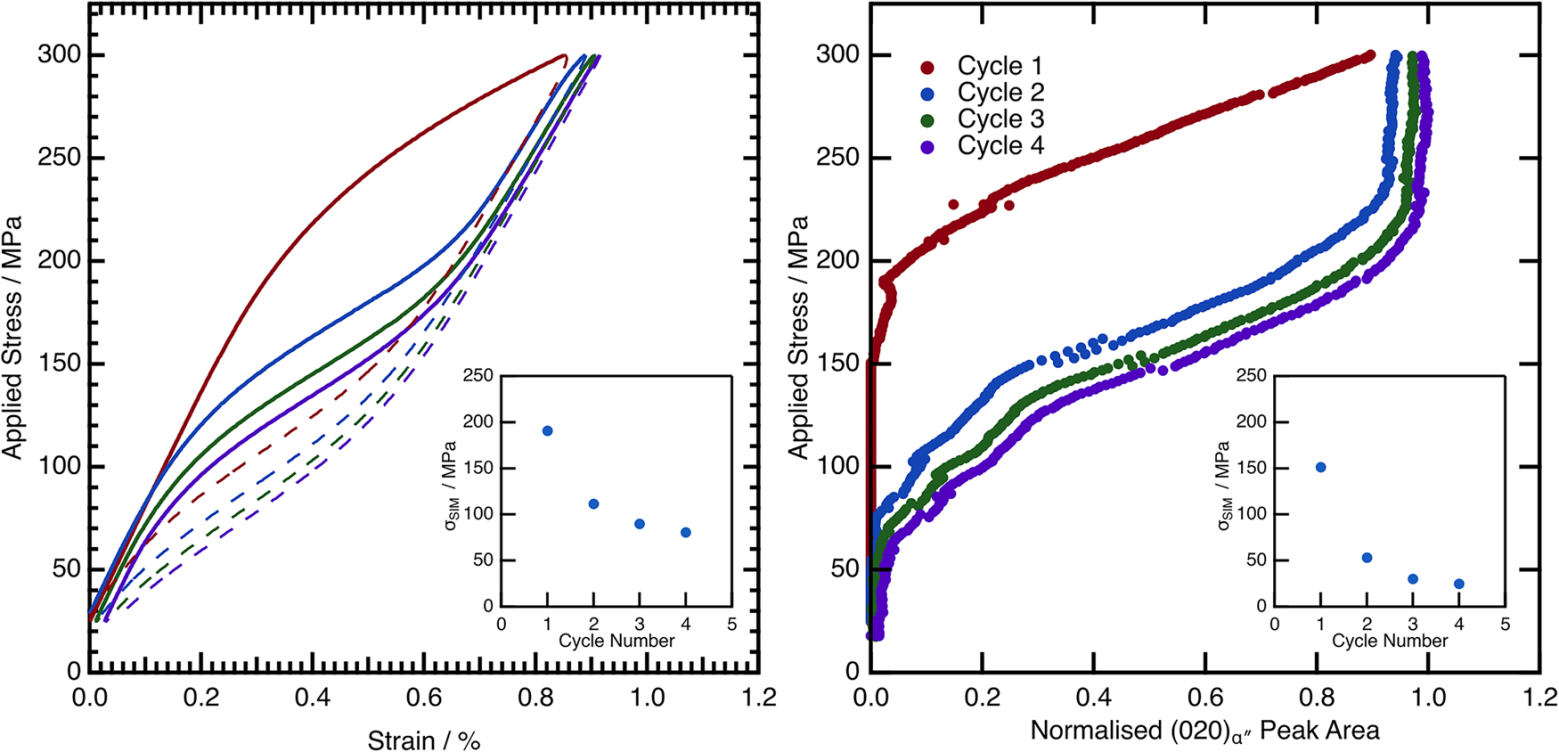
Cyclic test for the AC condition material to a fixed peak stress of 300 MPa. The insets show the value of σ_SIM_ measured from both the ex situ (left) and in situ (right) data. The ex situ data show the macroscopic stress-strain response of the material. The in situ data highlights the α″ peak area evolution from the diffraction data with the applied stress. The first two cycles of these data were previously published in ref. ^40^.

To investigate the degradation in functional properties further, a fresh sample in the AC condition was mechanically cycled both ex situ and in situ to a fixed peak stress of 300 MPa, which was shown to be a fully recoverable cycle. These data are shown in Fig. 13. In the first cycle, the sample initially behaves in a linear-elastic manner up to a stress of ~150 MPa as before. At this stress, there is a deviation from linear behaviour and an increase in the volume fraction of α″. On unloading from this first loading cycle, all of the strain and α″ is recovered. Upon loading in the second cycle, both the strain and α″ volume fraction begin to increase at a lower applied stress. Interestingly, once a comparable volume fraction of α″ is formed in this cycle, there appears to be largely linear-elastic deformation of the microstructure up to the peak stress. On unloading, there is a very small retention of α″ and strain in the microstructure.

The insets to each figure show the calculated σ_SIM_ values. Both show an initial rapid decrease in the value of σ_SIM_, before it continues to decrease at a much slower rate.

Of note in the tests performed so far is the observation that the transformation to α″ (either mechanically induced on loading or as a result of the rapid quench) acts to increase the stability of the α″, manifesting as a decreased σ_SIM_ or increased *M*_s_. However, heating had the opposite effect, whereby the stability of the α″ was reduced, with this effect more pronounced when heating to higher temperatures.

**Fig. 14:**
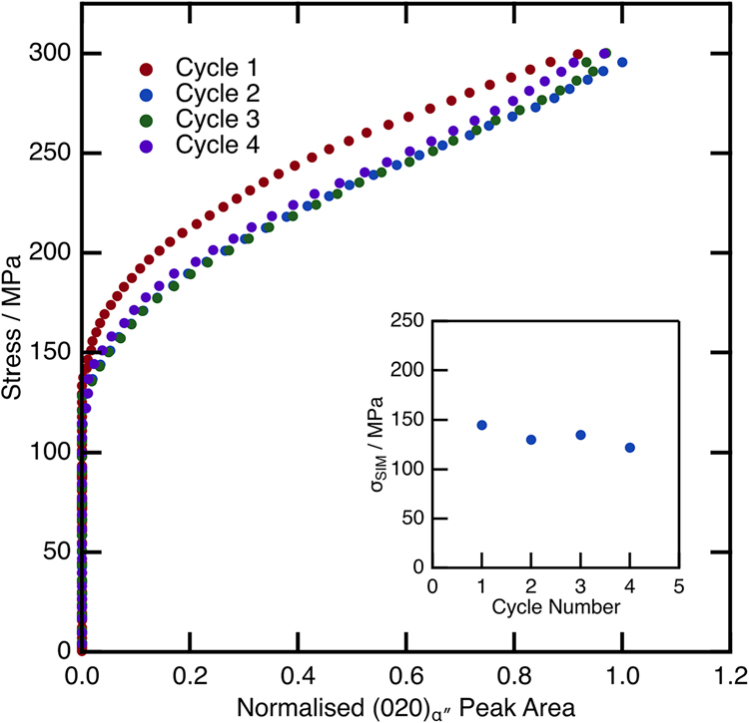
Cyclic tests for the AC material with an intercycle recovery heat treatment to 350 °C. For clarity, only the loading data is shown. The inset highlights the measured σ_SIM_ values from these data.

The cyclic test to a fixed applied stress was therefore repeated in situ with an intercycle heat treatment to 350 °C, to ascertain whether the increase in stability of the α″ following a load cycle could be reversed. These data are presented in Fig. 14.

The α″ peak area begins to evolve from ~150 MPa in the first cycle, which is consistent with the other tests performed. The sample was unloaded, and then heated and cooled. On a second cycle, there is only a minimal change in the value of σ_SIM_, which contrasts with the behaviour observed in Fig. 13. This shows that the heat treatment was effective at largely reversing the changes from the mechanical cycle, resulting in more stable cyclic loading behaviour.

**Fig. 15:**
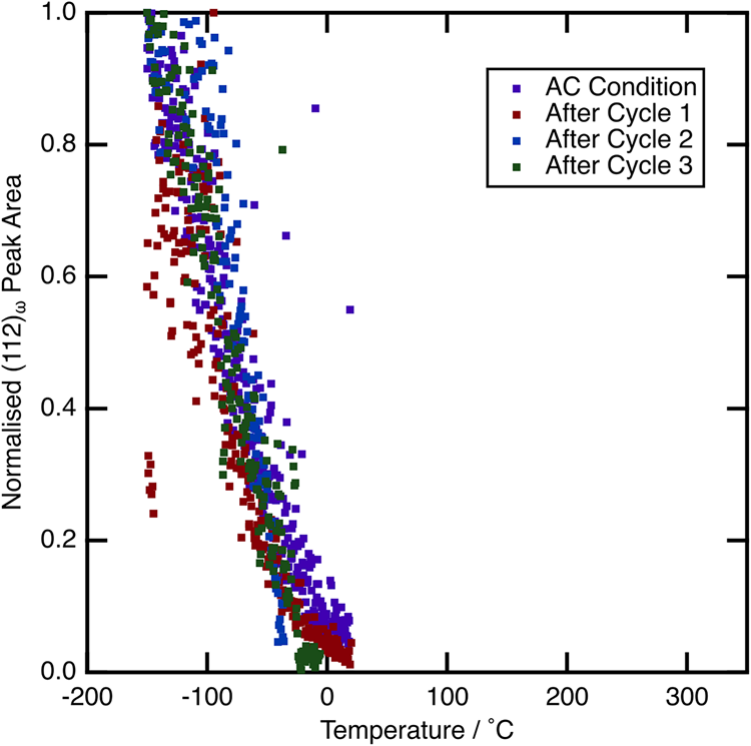
The evolution of the (112)_⍵_ peak area as a function of temperature, when cooling from 350 °C. Data shown is for the initial condition of the AC sample and on cooling from each intercycle heat treatment (which was applied following each mechanical cycle). Areas have been normalised to the value at −150 °C in each case. The data shown is for the original AC condition, and during the heat-cool treatment performed after each mechanical cycle.

As before, the possibility that the ⍵ phase might be responsible was considered. Again, no formation of ⍵_iso_ was observed when heating the sample, in any of the thermal cycles. Figure 15 shows the evolution of ⍵_ath_ on cooling in each thermal cycle, with these data compared to the initial data in the AC condition, originally presented in Fig. 6. This enables the identification of ⍵_s_ in each case. As can be seen, the evolution of ⍵_ath_ is identical in each cycle, with the same ⍵_s_ temperature. As such, ⍵_ath_ cannot be responsible for the changes seen in the mechanical data due to the thermal cycles.

## Discussion

By varying the cooling rate from the β phase field, two different microstructures could be obtained for an identical alloy composition. Quenching was shown to promote a transformation to α″, whilst cooling slowly retained a metastable β microstructure (Fig. 3). This has important implications for the inferred *M*_s_ temperature of the alloy. The room temperature was above *M*_s_ for the AC condition, but below *M*_s_ for the WQ condition.

Historically, variations in the ⍵ phase fraction between samples, as a result of different thermal histories, have been considered responsible for both discrepancies in transformation parameters (*M*_s_ and σ_SIM_) between samples and an evolution in these parameters with thermal cycling. For example, it has been reported that α″ and ⍵ can compete on cooling, with this competition having a pronounced rate dependence^43^. Although ⍵_ath_ is seen to form on cooling (Fig. 6), the ⍵_s_ temperature is invariant between samples, regardless of cooling rate from above the β transus temperature. Furthermore, previous work has highlighted that quenched specimens can still retain a metastable β microstructure close to the sample surface, whilst still transforming to α″ in the bulk^44^. If ⍵_iso_ formation were the cause of this change in microstructure, it may be expected that ⍵_iso_ formation would be greatest in the sample centre, where the cooling rate is slowest. However, the microstructural observations in the aforementioned work do not support this.

To generate this difference in behaviour on rapidly cooling the sample, it is necessary that there is a driving force for the formation of α″, which is not dependent on composition or the absolute temperature of the sample. Considering the Clausius–Clapeyron relationship^45,46^:

$$\frac{d{\sigma }_{{SIM}}}{{dT}}=\frac{{dS\; \rho }}{d\varepsilon }$$

this driving force can be supplied by either a change in temperature, *T*, or by applying a critical stress, σ_SIM_, with the entropy change between the β and martensite structures, ${dS}$, the density of the alloy, $\rho$, and the transformation strain between β and α″, $d\varepsilon$, remaining approximately constant. The applied stress must contain a component of shear to induce the transformation, but as these samples are polycrystalline, the resolved shear stress on favourably oriented planes will scale with the macroscopically applied load.

As such, by holding a sample under an applied load whilst heating and cooling, the measured *M*_s_ temperature is elevated^47,48^, similar to the elevated value seen here during a WQ versus an AC. However, in the present case, instead of a macroscopically applied load which would induce a macroscopic, or Type I strain, in the material, the strains present are inter- (Type II) or intra- (Type III) granular in nature. These types of strains are known to exist in engineering materials following cooling as a result of residual stresses in the material^49,50^. Furthermore, these strains often result in component distortion, indicating that they have a deviatoric component. It is also well reported that the residual stress levels are expected to be higher when rapidly cooling a sample, compared to when cooling more slowly^51,52^.

*M*_s_ was also shown to be affected by heating and cooling (Fig. 7), highlighting that the microstructure is dynamic. Short heating and cooling steps were able to depress *M*_s_ to lower temperatures, indicating an increase in the stability of the β phase. This effect was more pronounced when heating to higher temperatures, with the highest temperature of 350 °C depressing *M*_s_ to below −150 °C. This suggests that the behaviour of the WQ condition following heating is tending towards the behaviour of the AC condition and “reversing” the effects of the initial quench. However, the evolution of the α″ volume fractions on cooling suggests the *M*_s_ temperature is not just reduced, but the range of temperatures over which martensite is able to form is decreasing.

The transformation to α″ in the Ti2448 WQ sample will necessarily coincide with an increase in dislocation density, as dislocations are required to maintain geometric continuity at the phase boundary^53,54^. Dislocations can have both normal and shear components of stress and are responsible for many Type III strains within materials. Furthermore, they are known to be thermally activated, and given a sufficient driving force, will redistribute to lower the overall strain energy of the system^55^. The relaxation of these intragranular Type III strains would remove some of the associated driving force for the martensitic transformation when cooling the sample, resulting in a decrease in *M*_s_. This effect will be more pronounced when heating to higher temperatures, where dislocation activation is greatest. Redistribution of interfacial dislocations has been shown by TEM studies in Ti2448 when heating to these temperatures^38^, and has been correlated to subsequent changes in microstructure.

**Fig. 16:**
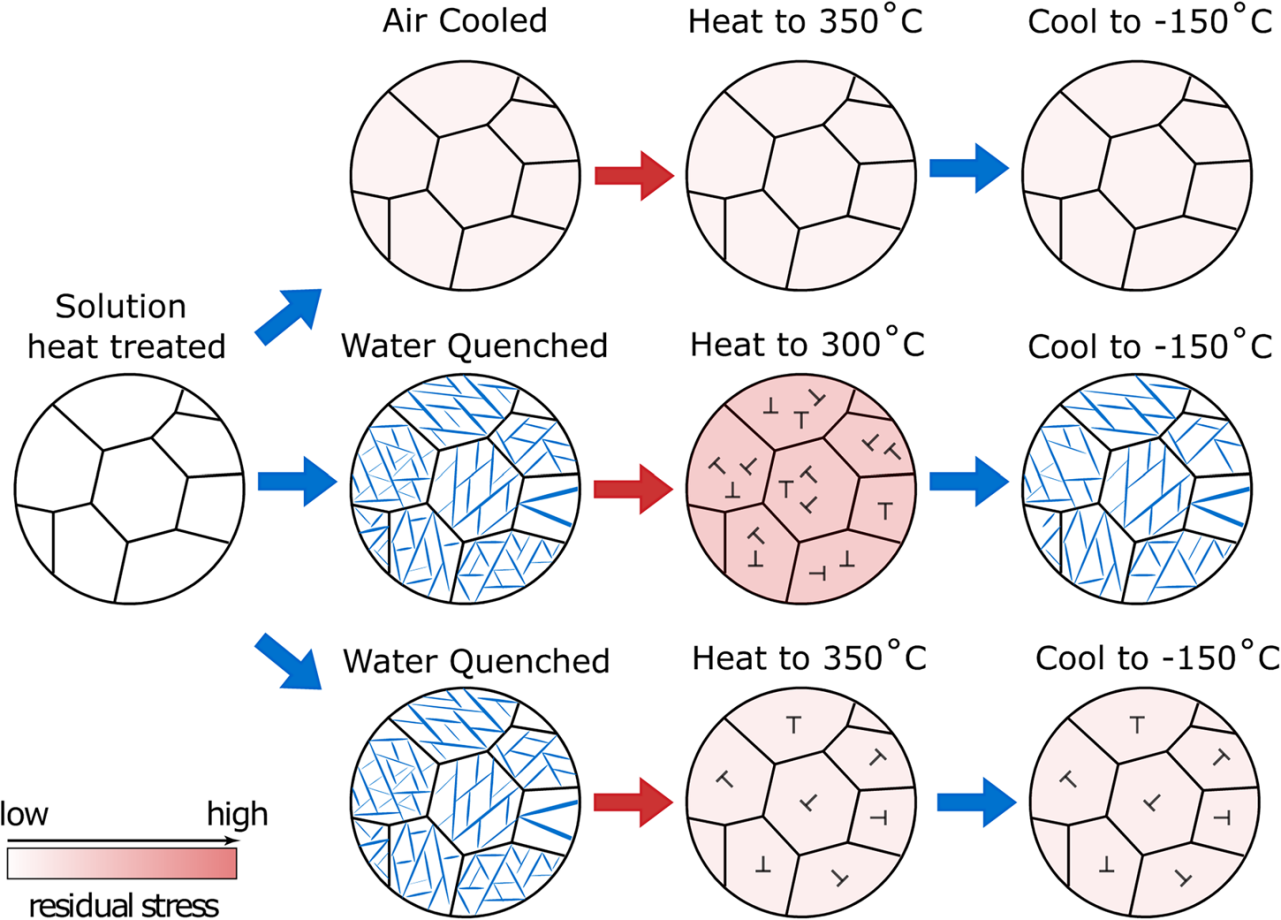
A schematic diagram highlighting the effect of different heat treatment conditions on the transformation response. The effect of different heat treatment conditions on the residual stress level is shown by the red-gradient colour bar. The effect of these different residual stress levels on the *M*_s_ temperature is shown.

To summarise how the initial cooling, and subsequent heat treatment influence the observed microstructure, and therefore the *M*_s_ temperature, of the alloys, the following schematic diagram has been produced (Fig. 16).

This has important implications, as aerospace industrial processes drive towards the miniaturisation of components. In WQ samples, we have previously made observations of a layer free from α″ at the surface of the sample^44^. Subsequent investigations highlighted that thin samples may be prevented from transforming to α″ completely. Cross-comparing literature data supports this observation^30,56,57^, with the resulting conclusion that thinner samples, or the surface of thicker samples, exhibit different *M*_s_ values than the centre of bulk specimens. This can be attributed to stress relaxation at a free surface following quenching and highlights further design criteria that must be considered when tailoring the thermal transformation behaviour to a given application.

However, the value of *M*_s_ can also be shown to influence the subsequent mechanical behaviour, even when testing samples with a nominally identical single phase microstructure, for example the AC and WQ + 300 °C conditions, shown in Fig. 9. The data presented in Fig. 12, have conclusively shown that ⍵_iso_ does not form during these thermal profiles of the experiments performed in this study, requiring at least 7 min at 300 °C before the onset of precipitation. As such, there must be an alternative mechanism responsible for the difference in transformation behaviour of the samples.

As an alternative, we propose that the different *M*_s_ temperature between the AC sample (*M*_s_ < −150 °C) and the WQ sample heated to 300 °C (*M*_s_ ~ −10 °C) are a consequence of very different residual stress levels. The AC material with the lower residual stress level needs to have a macroscopically applied load of ~150 MPa in order to induce the α″ martensite. In contrast, the WQ + 300 °C sample with a higher residual stress level only needs ~80 MPa of applied load in order to drive the same transformation.

Perhaps the more important aspects are the implications for the cyclic loading behaviour, whereby alloys exhibit a decrease in σ_SIM_ and in hysteresis area and concomitant decrease in vibration damping and actuation capabilities. Despite disparate initial transformation stresses, the value of σ_SIM_ decays at different rates. After only a few cycles, both conditions exhibit a σ_SIM_ that tends towards similar values. This tendency to converge on similar behaviour is even more apparent when considering the reversibility of the transformation. The AC condition is fully reversible on unloading to lower applied stresses, whereas the WQ + 300 °C condition accumulates residual α″ in the microstructure even on the first cycle. However, at higher applied loads, the AC condition also begins to accumulate some residual α″ in the microstructure. Following the final cycle to 450 MPa, the residual strain is comparable between the conditions. These data cannot be rationalised by considering a change in the precipitation of either ⍵_ath_ or ⍵_iso_, as the test temperature remains constant during the mechanical cycles, and both samples reach identical applied loads. Instead, the changes occurring in the material during mechanical cycling, which act to reduce σ_SIM_ and hence raise *M*_s_, must necessarily be equivalent, but opposite to the changes that occur during thermal cycling.

**Fig. 17:**
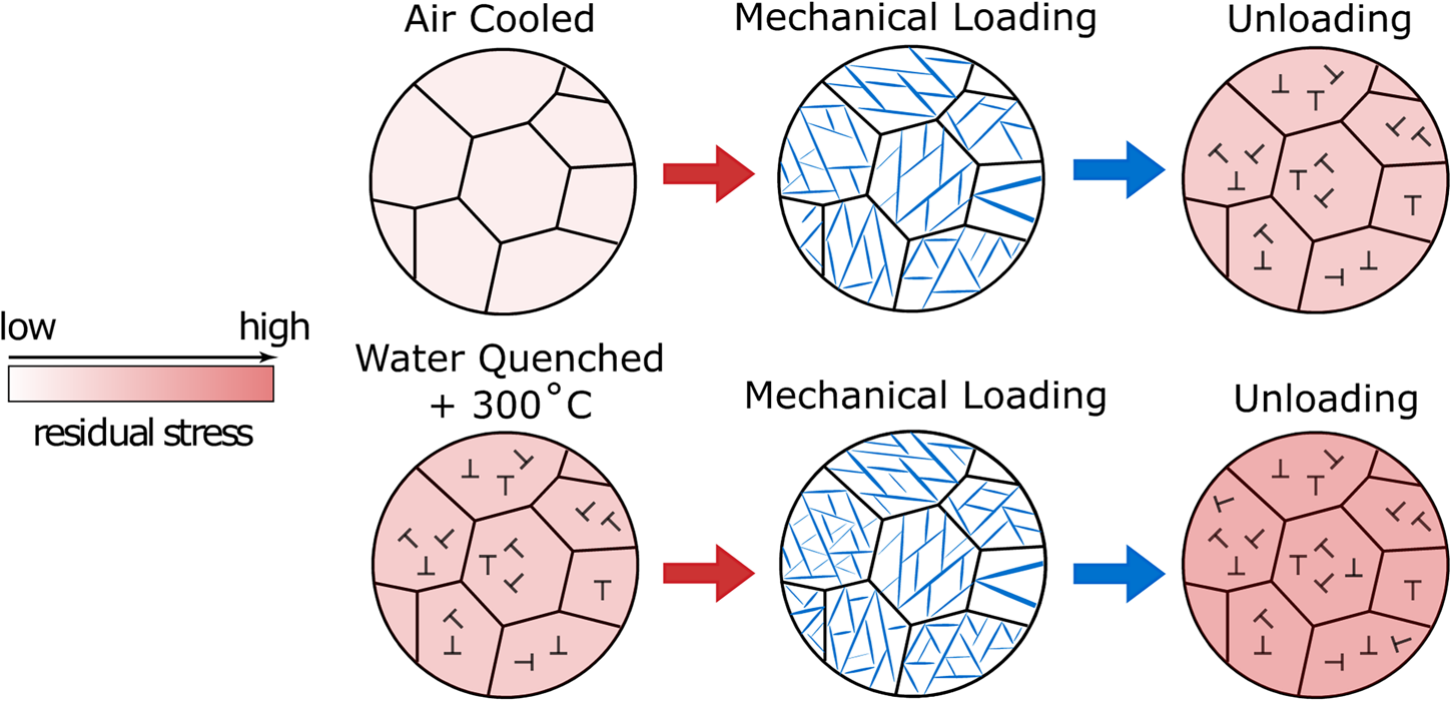
A schematic diagram highlighting the effect of residual stress levels on the subsequent mechanical response. The residual stress level is shown by the red-gradient colour bar.

As mentioned above, the formation of α″ on loading will necessarily result in the formation of interfacial dislocations to maintain geometric continuity at the parent/martensite phase boundary^53^. These will remain in the microstructure on unloading, even if the transformation is fully reversible. These dislocations have an associated strain field that can influence subsequent deformation behaviour. This additional driving force for the transformation manifests as a decrease in the macroscopically applied Type I stress needed to form α″ during the second loading cycle, and an associated increase in the *M*_s_ temperature. This principle is shown schematically in Fig. 17. Eventually, this effect is sufficient to increase the *M*_s_ temperature to above the testing temperature, retaining α″ even once the externally applied load has been removed.

Therefore, not only can the influence of residual stress in the microstructure rationalise the discrepancies in transformation stress observed in the literature, but it also provides a mechanism for cyclic degradation behaviour.

The possibility of reducing or preventing cyclic degradation by improving the compatibility of the parent and martensite phases at the interface has been considered within the literature^58–63^. The compatibility between the two phases can be measured by considering the eigenvalues of the transformation stretch tensor, which represent the principal distortions on the transformation. These are a function of the parent and martensite lattice parameters and so can be readily tuned by altering alloy composition. To maintain an invariant plane, one of these eigenvalues must equal unity, with the principal strain in this direction vanishing^53^. When it is the middle eigenvalue, λ_2_, that is unity, this represents a rational interface between the parent and martensite phases, which does not require the presence of dislocations to maintain compatibility.

In ordered systems, a positive correlation has been shown between λ_2_ and the extent of mechanical cyclic degradation, with alloys for which λ_2_ ~ 1 showing almost no changes in σ_SIM_ between subsequent cycles^58,60^. Whilst this concept can readily be extended to disordered transforming systems, such as metastable β alloys, our theory goes further. Not only are the dislocations associated with the transformation interface important, but all sources of residual stress can have an effect on the transformation behaviour. Here, we have shown that this could include stresses that are imparted on the material during quenching, but would also involve other sources of residual strain, provided there is some deviatoric component. This could be the strain fields associated with precipitates, complex geometries, grain boundaries, or even naturally occurring defects within the microstructure. This is consistent with reports of martensite initiating close to such features^64–67^ and reports of the martensitic transformation being influenced by dislocation slip in favourably oriented grains^68^. The martensite itself can also generate stress fields within the microstructure if there is an associated volume change during the transformation. As such, an additional criterion for good transformation reversibility is for the determinant of the transformation stretch tensor to equal unity, representing no volume change and hence no far field stresses^61^. This highlights the importance of understanding both the defect structure and the nature of stress distributions within a material when tuning the transformation behaviour of an alloy, even if λ_2_ = 1 can be achieved.

The λ_2_ = 1 condition has also been shown to correlate very strongly with narrow thermal and mechanical hysteresis^69–72^. This may not always be beneficial, as in some potential aerospace applications, a wide hysteresis is the very property that makes this class of alloy attractive. Furthermore, other sources of residual stress accumulation in a material would also be expected to contribute to functional fatigue behaviour. For example, dislocation generation is also known to occur during structural fatigue of ductile components, which could result in a change in residual stresses^73^. This could limit the effectiveness of alloy design strategies based solely on achieving a λ_2_ = 1. Instead, other methods may be needed in order to mitigate cyclic changes in behaviour.

One possible option for aerospace materials is a short “refresh”, whereby components are heated to relieve and redistribute residual stresses. In this study, a short intercycle heat and cool to 350 °C was shown to be effective at recovering σ_SIM_ to values prior to any deformation (Fig. 14). This could be employed alongside a higher interface compatibility (λ_2_ approaching 1), to achieve a balance of desirable hysteresis and tolerable cyclic degradation between refresh cycles.

However, at the current stage, the theory presented only gives a holistic view of the mechanisms underpinning the transformation. Many residual stresses within real components are heterogeneous in nature, and as such, the transformation will likely initiate in specific locations. This inherently introduces some issues when considering how to study the transformation further. To provide site-specific information, studies will typically be confined to surface techniques, which will not be representative of bulk behaviour due to the lack of constraint at a free surface.

## Conclusion

Discrepancies in key transformation-related parameters can be observed within the literature across a range of metastable β Ti–Nb alloys. Typically, this variability is ascribed to the ⍵ phase. However, we have provided evidence to suggest that neither ⍵_ath_ nor ⍵_iso_ can account for all of the observed phenomena. Instead, we have used a combination of in situ and ex situ techniques to show the importance of residual stresses on both the thermal and mechanical transformation.

In this study, significant residual stresses from rapidly quenching a sample were shown to promote a martensitic microstructure. Importantly, this microstructure was found to be dynamic, with subsequent heating and cooling resulting in changes in *M*_s_ that were more dramatic when heated to higher temperatures, as the residual stresses relax, and defects annihilate.

This was shown to have important consequences for the mechanical behaviour, with very different initial σ_SIM_ values observed for samples of identical starting microstructure but with differing residual stress levels. This demonstrated how the transformation is governed by the combination of macroscopically applied load and the contribution from inter- and intra-granular stresses.

After a number of mechanical cycles, the behaviour of the two alloy conditions was shown to be less disparate, with σ_SIM_ approaching comparable values. It is known that interfacial dislocations are introduced to accommodate the β/α″ phase boundary, and it is suggested that this increases residual stress levels to similar values in both conditions. However, by heating the tensile samples to temperatures of only 350 °C, and consequently lowering the residual stress levels, it was shown that functional fatigue behaviour could be reversed. This highlights important new methods by which component lifetimes could be improved, whilst maintaining desirable hysteretic properties.

Consequently, this work has served as a proof of concept that the total combination of external and residual stresses is critical for governing the transformation behaviour of this class of alloy. Crucially, it has also demonstrated a method by which the cyclic degradation in transformation properties can be managed. This enhanced mechanistic understanding directly addresses some of the key technological barriers for these materials and should enable greater industrial utilisation.

## Methods

Material for this study was taken from a commercially produced plate of Ti2448 with a measured composition of Ti–24.1Nb–3.92Zr–7.85Sn–0.078O–0.008 N wt%. It should be noted that this material has slightly lower O and Sn contents compared to other studies on Ti2448^29^ and, as such, the β phase stability is expected to be lower with respect to the α″ martensite.

Samples were taken from sections of the plate material that had been rolled to 0.6 mm in thickness so as to ensure even heating and cooling throughout the samples. Tensile samples with a gauge width of 0.6 mm were produced by electro-discharge machining (EDM) and lightly ground to 0.5 mm × 0.5 mm final cross-section to remove the recast layer. Samples were then sealed in quartz ampoules, with a Ta getter. The ampoules were evacuated and backfilled with Ar three times, before a final evacuation to ~5 × 10^−5^ mbar. Samples were then solution heat-treated at 900 °C for 5 min before cooling. Some samples were AC slowly within the ampoules. Other samples were rapidly cooled via water quenching (WQ) by breaking the ampoule under ice-water.

Specimens were prepared for metallographic examination by grinding using fine SiC paper before polishing with a 0.04 μm colloidal silica suspension, buffered with H_2_O_2_ to pH 7. Importantly, samples were not mounted during this process to prevent any thermally induced effects from the mounting process, as even cold mounting solutions exhibit an exothermic reaction during curing, which can alter thermally sensitive microstructures. Scanning electron microscopy (SEM) was performed on a Zeiss GeminiSEM 300 operated at 15 kV with a 60 μm aperture. Images were obtained using back-scattered electrons (BSE) with a short working distance to optimise channelling contrast.

Mechanical testing was performed both ex situ and in situ to assess cyclic changes in behaviour. Ex situ mechanical testing was performed on an Instron 3367B test frame with a 30 kN load cell and a 12.5 mm Epsilon contact extensometer. Loading was performed in stress control at a rate of 4 MPa s^−1^, which is in a quasistatic testing regime for this alloy type. Samples were first pre-loaded to 25 MPa, which is within ASTM E8 guidelines for tensile testing, and then cyclically loaded^74^. Incremental stress tests were performed on both sample conditions, in increments of 50 MPa. Cyclic loading to a fixed peak stress of 300 MPa was performed on the AC condition material only. σ_SIM_ was determined by fitting a line to the linear-elastic region of each cycle, offsetting this line by 0.02%, and taking the intersection of this line with the cyclic data.

In situ mechanical testing was performed on the I12 beamline at Diamond Light Source using a Linkam TST350 operated in displacement control at 4 μm s^−1^. Loading profiles were selected to match the tests performed ex situ. Two-dimensional diffraction patterns were collected during loading with an exposure time of 1 s using a CdTe Pilatus detector on the I12 beamline at Diamond Light Source^75^. The beam energy was calibrated with a CeO2 NIST standard at multiple sample to detector distances^76^. 2D data was reduced to 1D using DAWN software by a 360° azimuthal integration^77,78^. The diffraction peaks were fitted with Gaussian functions on a linear background using Wavemetrics Igor Pro, with peak area taken as a proxy for phase volume fraction.

Thermal cycling, including intercycle heat treatments, was performed in situ in the same Linkam TST350 sample environment. Heating and cooling were achieved at 25 °C min^−1^ with diffraction data collected continuously with a 2 s exposure time using the same experimental methodology as above. An additional 8 h isothermal age at 300° °C was performed using the same heating and cooling rate and data acquisition parameters in a Linkam 1500 V furnace.

A focused ion beam (FIB) lamella was prepared for TEM using an FEI Helios Ga Ion FIB. A Pt protective layer was deposited onto the sample surface, and a 5 μm deep trench was milled in a region of interest using 9 nA 10 kV 188 Ga ion beam. The samples were undercut and transferred to a Cu grid using an omni probe. Samples were milled to electron transparency using sequentially lower beam currents. Finally, a low kV ion polish was utilised.

TEM data were collected on a Thermo Fisher Scientific Spectra fitted with a Quantum Detectors Merlin Detector. Scanning Electron Diffraction (SED) data were obtained using a convergence angle of 0.5 mrad, a beam current of 40 pA, and a beam diameter of 4 nm. These data were then processed in the HyperSpy software package to generate virtual dark field (DF) images from specific regions of diffracted intensity.

Scanning TEM (STEM) energy dispersive spectroscopy data were collected using 4 EDS Super-X detectors, over the same area of the sample, to identify any composition inhomogeneity. A beam current of 100 pA, a convergence angle of 24 mrad, a collection angle of 101–200 mrad, and a condenser aperture of 70 μm were used.

## Data Availability

The underlying research data required to reproduce these findings are available from the University of Cambridge repository (10.17863/CAM.110909).

## References

[1] Lutjering, G. & Williams, J. *Titanium* (Springer Berlin, Heidelberg, 2007); 10.1007/978-3-540-73036-1

[2] Kolli, R. P. & Deveraj, A. A review of metastable beta titanium alloys. *Metals* **8**, 1–41 (2018).

[3] Peters, M., Kumpfert, J., Ward, C. H. & Leyens, C. Titanium alloys for aerospace applications. *Adv. Eng. Mater.* **5**, 419–427 (2003).

[4] Boyer, R. R. Titanium for aerospace: rationale and applications. *Adv. Perform. Mater.* **2**, 349–368 (1995).

[5] Lee, G. C., Liang, Z., Gan, Q. & Niu, T. *Vibration Reduction of Helicopter Blade Using Variable Dampers—a Feasibility Study* (University of New York, Buffalo, 2002).

[6] Mohd Jani, J., Leary, M., Subic, A. & Gibson, M. A. A review of shape memory alloy research, applications and opportunities. *Mater. Des.* **56**, 1078–1113 (2014).

[7] Bankar, V. K. & Aradhye, A. S. A review on active, semi-active and passive vibration damping. *Int. J. Curr. Eng. Technol.* **6**, 2187–2191 (2016).

[8] Hartl, D. J. & Lagoudas, D. C. Aerospace applications of shape memory alloys. *Proc. Inst. Mech. Eng. J. Aerosp. Eng.* **221**, 535–552 (2007).

[9] Kim, H. Y. & Miyazaki, S. Martensitic transformation and superelastic properties of Ti–Nb base alloys. *Mater. Trans.* **56**, 625–634 (2015).

[10] McMahon, R. E. et al. A comparative study of the cytotoxicity and corrosion resistance of nickel–titanium and titanium–niobium shape memory alloys. *Acta Biomater.* **8**, 2863–2870 (2012). 22465573 10.1016/j.actbio.2012.03.034

[11] Frenzel, J. et al. Influence of Ni on martensitic phase transformations in NiTi shape memory alloys. *Acta Mater.* **58**, 3444–3458 (2010).

[12] Kim, H. Y. & Miyazaki, S. *Ni-Free Ti-Based**Shape Memory Alloys* (Elsevier, 2018).

[13] Duerig, T. W., Melton, K. N., Stockel, D. & Wayman, C. M. *Engineering Aspects of Shape Memory Alloys* (Elsevier, 1990); 10.1016/C2013-0-04566-5.

[14] Froes, F. H. & Bomberger, H. B. The beta titanium alloys. *JOM* **37**, 28–37 (1985).

[15] Sadiq, H., Wong, M. B., Al-Mahaidi, R. & Zhao, X. L. The effects of heat treatment on the recovery stresses of shape memory alloys. *Smart Mater. Struct.* **19**, 035021 (2010).

[16] Matsumoto, H., Watanabe, S. & Hanada, S. Beta TiNbSn alloys with low Young’s modulus and high strength. *Mater. Trans.* **46**, 1070–1078 (2005).

[17] Church, N. L., Talbot, C. E. P., Connor, L. D., Michalik, S. & Jones, N. G. The interdependence of the thermal and mechanical cycling behaviour in Ti2448 (Ti–24Nb–4Zr–8Sn, wt%). *Mater. Sci. Eng. A* **899**, 145791 (2024).

[18] Pang, E. L., Hildyard, E. M., Connor, L. D., Pickering, E. J. & Jones, N. G. The effect of quench rate on the β-α’’ martensitic transformation in Ti–Nb alloys. *Mater. Sci. Eng. A* **817**, 141240 (2021).

[19] Li, S. J., Jia, M. T., Prima, F., Hao, Y. L. & Yang, R. Improvements in nonlinear elasticity and strength by grain refinement in a titanium alloy with high oxygen content. *Scr. Mater.* **64**, 1015–1018 (2011).

[20] Yang, Y. et al. Characterization of the martensitic transformation in the superelastic Ti–24Nb–4Zr–8Sn alloy by in situ synchrotron X-ray diffraction and dynamic mechanical analysis. *Acta Mater.* **88**, 25–33 (2015).

[21] Coakley, J. et al. Microstructural evolution in a superelastic metastable beta-Ti alloy. *Scr. Mater.* **128**, 87–90 (2017).

[22] Hildyard, E. M. et al. The influence of microstructural condition on the phase transformations in Ti–24Nb (at.%). *Acta Mater.* **199**, 129–140 (2020).

[23] Bönisch, M. et al. Thermal stability and latent heat of Nb-rich martensitic Ti–Nb alloys. *J. Alloy. Compd.* **697**, 300–309 (2017).

[24] Al-Zain, Y., Kim, H. Y., Hosoda, H., Nam, T. H. & Miyazaki, S. Shape memory properties of Ti–Nb–Mo biomedical alloys. *Acta Mater.* **58**, 4212–4223 (2010).

[25] Ahmed, T. & Rack, H. J. Martensitic transformations in Ti-(16–26 at %) Nb alloys. *J. Mater. Sci.* **31**, 4267–4276 (1996).

[26] Kim, H. Y., Ohmatsu, Y., Kim, J. I., Hosoda, H. & Miyazaki, S. Mechanical properties and shape memory behavior of Ti–Nb alloys. *Mater. Trans.* **45**, 1090–1095 (2004).

[27] Wang, H. L. et al. Elastically confined martensitic transformation at the nano-scale in a multifunctional titanium alloy. *Acta Mater.* **135**, 330–339 (2017).

[28] Héraud, L., Castany, P., Ijaz, M. F., Gordin, D. M. & Gloriant, T. Large-strain functional fatigue properties of superelastic metastable β titanium and NiTi alloys: a comparative study. *J. Alloy. Compd.* **953**, 170170 (2023).

[29] Hao, Y. L., Li, S. J., Sun, S. Y., Zheng, C. Y. & Yang, R. Elastic deformation behaviour of Ti–24Nb–4Zr–7.9Sn for biomedical applications. *Acta Biomater.* **3**, 277–286 (2007). 17234466 10.1016/j.actbio.2006.11.002

[30] Coakley, J., Rahman, K. M., Vorontsov, V. A., Ohnuma, M. & Dye, D. Effect of precipitation on mechanical properties in the β-Ti alloy Ti–24Nb–4Zr–8Sn. *Mater. Sci. Eng. A* **655**, 399–407 (2016).

[31] Qian, M. F., Zhang, X. X., Witherspoon, C., Sun, J. F. & Müllner, P. Superelasticity and shape memory effects in polycrystalline Ni–Mn–Ga microwires. *J. Alloy. Compd.* **577S**, S296–S299 (2013).

[32] López-Ferreño, I. et al. High-temperature shape memory alloys based on the Cu–Al–Ni system: design and thermomechanical characterization. *J. Mater. Res. Technol.* **9**, 9972–9984 (2020).

[33] Reed, O. G., Church, N. L. & Jones, N. G. Understanding the room temperature recovery behavior in a Ti–24Nb–4Zr–8Sn superelastic alloy. *Adv. Eng. Mater.* **26**, 2400076 (2024).

[34] Church, N. L., Ang, J. Y., Reed, O. G., Gibson, O. E. & Jones, N. G. The effect of athermal omega on the transformation behaviour of Ti–24Nb–4Zr–8Sn (wt%). *Materialia* **38**, 102266 (2024).

[35] Li, S., Choi, M.-s & Nam, T.-h. Role of fine nano-scaled isothermal omega phase on the mechanical and superelastic properties of a high Zr-containing Ti–Zr–Nb–Sn shape memory alloy. *Mater. Sci. Eng. A* **782**, 139278 (2020).

[36] Okamoto, N. L. et al. Why is neutral tin addition necessary for biocompatible β-titanium alloys?—synergistic effects of suppressing ω transformations. *Acta Mater.* **273**, 119968 (2024).

[37] Church, N. L., Talbot, C. E. P. & Jones, N. G. On the influence of thermal history on the martensitic transformation in Ti–24Nb–4Zr–8Sn (wt%). *Shape Mem Superelasticity* **7**, 166–178 (2021).

[38] Church, N. et al. Evidence of dislocation dependent behaviour in superelastic Ti2448 (Ti–24Nb–4Zr–8Sn, wt%). *Acta Mater.* **255**, 119066 (2023).

[39] Church, N. L. & Jones, N. G. The influence of stress on subsequent superelastic behaviour in Ti2448 (Ti–24Nb–4Zr–8Sn, wt%). *Mater. Sci. Eng. A* **833**, 142530 (2022).

[40] Church, N., Talbot, C., Connor, L., Michalik, S. & Jones, N. Functional fatigue during superelastic load cycling of Ti2448 (Ti–24Nb–4Zr–8Sn, wt%). *Materialia* **28**, 101719 (2023).

[41] Hickman, B. S. The formation of omega phase in titanium and zirconium alloys: a review. *J. Mater. Sci.* **4**, 554–563 (1969).

[42] Chluba, C. et al. Effect of crystallographic compatibility and grain size on the functional fatigue of sputtered TiNiCuCo thin films. *Philos*. *Trans*. *A**Math*. *Phys*. *Eng*. *Sci*. **374**, 20150311 (2016). 10.1098/rsta.2015.0311 PMC4938070 27402935

[43] Moffat, D. L. & Larbalestier, D. C. Competition between martensite and omega in quenched TiNb alloys. *Metall. Trans. A* **19**, 1677–1686 (1988).

[44] Church, N., Connor, L. & Jones, N. The effect of sample size on the microstructure and mechanical properties of Ti2448 (Ti–24Nb–4Zr–8Sn wt%). *Scr. Mater.* **222**, 115035 (2023).

[45] Natalia, R. & Sergey, B. Entropy change in the B2→B19′ martensitic transformation in TiNi alloy. *Thermochim. Acta* **602**, 30–35 (2015).

[46] Grassi, E. N. D., Chagnon, G., de Oliveira, H. M. R. & Favier, D. Anisotropy and Clausius–Clapeyron relation for forward and reverse stress-induced martensitic transformations in polycrystalline NiTi thin walled tubes. *Mech. Mater.* **146**, 103392 (2020).

[47] Rodinò, S. et al. Development of an automated experimental system for thermomechanical and electrical characterization of NiTi shape memory alloys. *Exp. Mech.* **64**, 425–440 (2024).

[48] Jones, N. G. & Dye, D. Martensite evolution in a NiTi shape memory alloy when thermal cycling under an applied load. *Intermetallics* **19**, 1348–1358 (2011).

[49] Fan, M. et al. Effect of residual stress induced by different cooling methods in heat treatment on the fatigue crack propagation behaviour of GH4169 Disc. *Materials* **15**, 5228 (2022). 10.3390/ma15155228 PMC9369495 35955161

[50] Rai, J. K., Mishra, A. & Rao, U. R. K. Residual stresses due to quenching process. *Int. J. Mach. Tool. Des. Res.* **20**, 1–8 (1980).

[51] Zhan, Y., Xu, H., Du, W. & Liu, C. Research on the influence of heat treatment on residual stress of TC4 alloy produced by laser additive manufacturing based on laser ultrasonic technique. *Ultrasonics* **115**, 106466 (2021). 10.1016/j.ultras.2021.106466 34020226

[52] Takase, A., Ishimoto, T., Suganuma, R. & Nakano, T. Surface residual stress and phase stability in unstable β-type Ti–15Mo–5Zr–3Al alloy manufactured by laser and electron beam powder bed fusion technologies. *Addit. Manuf.* **47**, 102257 (2021).

[53] Lieberman, D. S., Wechsler, M. S. & Read, T. A. Cubic to orthorhombic diffusionless phase change—experimental and theoretical studies of AuCd. *J. Appl. Phys.* **26**, 473–484 (1955).

[54] Obbard, E. G. et al. The effect of oxygen on α” martensite and superelasticity in Ti–24Nb–4Zr–8Sn. *Acta Mater.* **59**, 112–125 (2011).

[55] Grabowski, B. & Zotov, N. Thermally-activated dislocation mobility in bcc metals: an accelerated molecular dynamics study. *Comput. Mater. Sci.* **200**, 110804 (2021).

[56] Duerig, T.W., Albrecht, J., Richter, D. & Fischer, P. Formation and reversion of stress induced martensite Ti–10V–2Fe–3Al. *Acta Metall. Mater* **30**, 2161–2172 (1982).

[57] Yang, Y. et al. Texture investigation of the superelastic Ti–24Nb–4Zr–8Sn alloy. *J. Alloy. Compd.* **591**, 85–90 (2014).

[58] König, D., Zarnetta, R., Savan, A., Brunken, H. & Ludwig, A. Phase transformation, structural and functional fatigue properties of Ti–Ni–Hf shape memory thin films. *Acta Mater.* **59**, 3267–3275 (2011).

[59] Xue, D., Li, Z., Pan, Y. & Zhang, G. Low hysteresis and high cyclic stability in a Ti50Ni45.2Cu1Fe3.8 shape memory alloy. *J. Alloys Compd.*10.1016/j.jallcom.2023.170188 (2023).

[60] Ahadi, A., Ghorabaei, A. S., Shirazi, H. & Nili-Ahmadabadi, M. Bulk NiTiCuCo shape memory alloys with ultra-high thermal and superelastic cyclic stability. *Scr. Mater.* **200**, 113899 (2021).

[61] James, R. D. & Zhang, Z. A way to search for multiferroic materials with “unlikely” combinations of physical properties. in *Magnetism and Structure in Functional Materials* (eds. Planes, A., Mañosa, L. & Saxena, A.) (Springer, 2004).

[62] Ball, J. M. & James, R. D. *Proposed Experimental Tests of a Theory of Fine Microstructure and the Two-Well Problem* (The Royal Society, 1992); https://royalsocietypublishing.org/.

[63] Shi, H., Delville, R., Srivastava, V., James, R. D. & Schryvers, D. Microstructural dependence on middle eigenvalue in TiNiAu. *J. Alloy. Compd.* **582**, 703–707 (2014).

[64] Acar, E. et al. Role of aging time on the microstructure and shape memory properties of NiTiHfPd single crystals. *Mater. Sci. Eng. A* **573**, 161–165 (2013).

[65] Ibarra, A., Caillard, D., San Juan, J. & Nó, M. L. Martensite nucleation on dislocations in Cu–Al–Ni shape memory alloys. *Appl Phys. Lett.* **90**, 101907 (2007).

[66] Song, T. & De Cooman, B. C. Martensite nucleation at grain boundaries containing intrinsic grain boundary dislocations. *ISIJ Int.* **54**, 2394–2403 (2014).

[67] Sidharth, R., Celebi, T. B. & Sehitoglu, H. Origins of functional fatigue and reversible transformation of precipitates in NiTi shape memory alloy. *Acta Mater.* **274**, 119990 (2024).

[68] Sedmák, P., Šittner, P., Pilch, J. & Curfs, C. Instability of cyclic superelastic deformation of NiTi investigated by synchrotron X-ray diffraction. *Acta Mater.* **94**, 257–270 (2015).

[69] Evirgen, A. et al. Relationship between crystallographic compatibility and thermal hysteresis in Ni-rich NiTiHf and NiTiZr high temperature shape memory alloys. *Acta Mater.* **121**, 374–383 (2016).

[70] Zarnetta, R. et al. Identification of quaternary shape memory alloys with near-zero thermal hysteresis and unprecedented functional stability. *Adv. Funct. Mater.* **20**, 1917–1923 (2010).

[71] Tong, Y., Shuitcev, A. & Zheng, Y. Recent development of TiNi-based shape memory alloys with high cycle stability and high transformation temperature. *Adv. Eng. Mater.* **22**, 1900496 (2020).

[72] Zhang, Z., James, R. D. & Müller, S. Energy barriers and hysteresis in martensitic phase transformations. *Acta Mater.* **57**, 4332–4352 (2009).

[73] Anderson, T. L. *Fracture Mechanics: Fundamentals and Applications* (CRC Press, 1995).

[74] ASTM International. ASTM E8/E8M standard test methods for tension testing of metallic materials. *Annual Book of ASTM Standards* 1–27 (ASTM, 2013); 10.1520/E0008.

[75] Drakopoulos, M. et al. I12: The joint engineering, environment and processing (JEEP) beamline at diamond light source. *J. Synchrotron. Radiat.* **22**, 828–838 (2015). 25931103 10.1107/S1600577515003513 PMC4416690

[76] Hart, M. L., Drakopoulos, M., Reinhard, C. & Connolley, T. Complete elliptical ring geometry provides energy and instrument calibration for synchrotron-based two-dimensional X-ray diffraction. *J. Appl. Crystallogr.* **46**, 1249–1260 (2013). 24068840 10.1107/S0021889813022437 PMC3778320

[77] Filik, J. et al. Processing two-dimensional X-ray diffraction and small-angle scattering data in DAWN 2. *J. Appl. Crystallogr.* **50**, 959–966 (2017). 28656043 10.1107/S1600576717004708 PMC5458597

[78] Basham, M. et al. Data analysis WorkbeNch (DAWN). *J. Synchrotr. Radiat.* **22**, 853–858 (2015). 10.1107/S1600577515002283 PMC4416692 25931106

